# Paradigm Shift: New Ideas for a Structural Approach to NCD Prevention: Comment on "How Neoliberalism Is Shaping the Supply of Unhealthy Commodities and What This Means for NCD Prevention"

**DOI:** 10.15171/ijhpm.2019.105

**Published:** 2019-11-09

**Authors:** Ashley Schram, Sharni Goldman

**Affiliations:** School of Regulation and Global Governance, Australian National University, Canberra, ACT, Australia.

**Keywords:** Non-communicable Diseases, Neoliberalism, Health Policy

## Abstract

It is a well-documented fact that transnational corporations engaged in the production and distribution of health-harmful commodities have been able to steer policy approaches to address the associated burden of non-communicable diseases (NCDs). While the political influence that corporations wield stems in part from significant financial resources, it has also been enabled and magnified by what has been referred to as global health’s neoliberal deep core, which has subjected health policy to the individualisation of risk and responsibility and the privileging of market-based policy responses. The accompanying perspective article from Lencucha and Thow draws attention to neoliberalism in the NCD space and the way it has historically structured patterns of thinking and doing that foreground economic interests over health considerations. In this commentary, we explore how shifting from a focus on material power to discursive power creates space to see the NCD agenda as a battle of economic ideas as well as dollars, and consequently the importance of public health engagement in the next vision for the economy.

Efforts to address the global burden of non-communicable disease (NCD) remain insufficient.^1^ Maintaining status quo behavioural interventions targeting smoking, alcohol, diet and physical activity will leave us short of meeting Target 3.4 of the Sustainable Development Goals to reduce premature mortality from NCDs by one third by 2030. Structural determinants of NCDs are widely acknowledged, such as the role of the private sector in regulation or the provision of a healthy living wage.^2^ Yet, guiding documents for how to tackle NCDs frequently undergo *lifestyle drift,* where recognition of structural causes transforms into recommended actions targeted at individual behaviours and lifestyle choices.^3^ One explanation for this drift has been the power of corporations to derail government measures that would reduce their profits. While corporate influence over health policy environments is well-documented,^4-6^ the story is more complex. The accompanying article from Lencucha and Thow lays out the argument that, “[c]ontrary to the assumption that policy-makers in these sectors are wilfully aligned with commercial interests, this perspective illustrates that government officials are often bound up within historically structured patterns of thinking and doing that foreground economic interests over health considerations” (p. 517).^7^

## Why Neoliberalism Is Incoherent With a Structural Approach to NCD Prevention?

Lencucha and Thow draw much needed attention to what has been referred to as global health’s *neoliberal deep core* which has subjected health policy “to the deployment and privileging of market-based policy responses, to commodification, privatisation, liberalisation of health and healthcare, and to the individualisation of risk and responsibility for health” (p. 163).^8^ This pattern of policy preferences can be hostile to NCD prevention efforts targeted at reducing markets in highly profitable products (eg, tobacco, alcohol and ultra-processed food and beverages).

The commodification of all aspects of society further renders the health-enhancing or health-diminishing properties of any good or service largely inconsequential. Ailments of consumption, such as obesity, diabetes or cancer, produce new markets of the sick. For example, the same companies that profit from making us fat, profit from making us thin (eg, Unilever’s purchase of Slimfast or Nestle’s purchase of Jenny Craig^9^). Whether the market is selling us sickness or health, sales contribute equally to the gross domestic product – the ultimate indicator of success within the neoliberal paradigm – thus allowing healthy economies to be underpinned by unhealthy societies.

Deference to the market is compounded by the neoliberal narrative of individual responsibility, which chafes at the suggestion that environments are a driving force of individual behaviour and would require some form of government intervention. The neoliberal rationality presumes and promotes self-governing subjects who should approach the body as a site of investment, wherein a healthy diet, avoidance of tobacco and alcohol, and physical activity represent good investment practices.^10^ Accordingly, structural action to meet the Sustainable Development Goal targets on NCDs under a neoliberal rationality will be an uphill battle. It has been said that “[y]ou never change things by fighting the existing reality. To change something, build a new model that makes the existing model obsolete” (p. 137).^11^ We believe it is time for more public health actors to join the debate over the next paradigm that will drive our economy and inevitably shape approaches within health policy.

## What Does a Structural Approach to NCD Prevention Need in a New Paradigm?

Drawing from the economic and regulatory literature, we propose three interrelated shifts needed in a new economic paradigm to support a more structural approach to reducing the global burden of NCDs. This is not intended to be a definitive list, but rather, is intended to start a conversation about economic paradigms among a broader public health audience. First, we believe the shift away from economic policy designed to grow the size of the economy, towards economic policy designed to reshape the economy will be necessary.^12^ As Denniss notes, intuitively people understand that having more of something, just for the sake of having more of it, is not desirable. Arguably, owning five pairs of shoes in your size is preferable to owning 10 pairs that are a size too small. Yet, we seem to overlook the distinction between quality and quantity when it comes to the economy. Greater attention to the non-monetary value of goods and services to us as individuals and a society would shift us away from the neoliberal tendency to commodify all aspects of our lives, which then prevents us from discerning between goods that support our well-being and goods that detract from it. This shift in mindset should provide greater public and political support for the regulation of health harmful commodities. For example, the type of exceptionalism we have seen within tobacco control could be spread to a greater range of health-harming goods and services.

Second, we propose a shift away from equating free markets with free choice, towards a more nuanced understanding of the multitude of institutional, ideational, cultural and social forces interacting to regulate our behaviour. Under neoliberalism, corporations have successfully framed their collection of techniques for influencing consumer taste, preferences and attitudes as enhancing choice, and state regulation of those techniques as intervening in that choice. A more honest narrative around choice – those who seek to shape it and to what end – would permit a less reductionist state versus market view. It would open space for a conversation about what we need in a regulatory approach in order to support informed choice within environments that (at the bare minimum) make healthy products and services equally affordable and accessible as unhealthy ones.

Finally, we propose that the next paradigm for the economy must embed development within an understanding of planetary boundaries. While planetary health is essential to human health writ large, environmentally-oriented economic policy would support expansion of the risk factors for NCDs to achieve more comprehensive change. At present, guidance on NCDs focuses on the ‘Big Four’ risk factors – tobacco, alcohol, diet and physical inactivity – which targets action towards individual behaviour change. Yet, we know that air pollution now kills more people annually than smoking,^13^ and that climate change is increasing rates of cardiovascular and respiratory diseases following increased intensity of heat extremes.^14^ Greater appreciation for human-planetary interactions and associated systems-approaches in the next paradigm could help put an end to the game of NCD risk factor ‘whack-a-mole’ we are currently playing and allow space to tackle more structural root causes.

## What Elements of a New Paradigm Currently Exist?

The seeds for a paradigm change are already in place – the question for public health will be how we identify, aggregate, and scale them up, and resit their co-optation by the neoliberal paradigm. The Kingdom of Bhutan, for example, has been using Gross National Happiness as an alternative to gross domestic product since 1972. In 2010, the US state of Maryland officially began reporting the Genuine Progress Indicator, a measure of the economy which factors in income inequality, household and volunteer work, higher education, and accounts for costs to the environment and society such as pollution, crime and unemployment. Since then, eight other states have followed suit with many more under consideration.^15^ In 2019, New Zealand announced the first ‘well-being budget’ designed to support long term impacts in the policy-making process, such as improving sustainability, lifting Māori and Pacific income, and reducing child poverty, rather than just short-term output measures.^16^

Research on ‘diverse economies’ – activities outside the mainstream economy such as cooperatives and collectives – is also elucidating how alternative models of exchange are sites of praxis for market-society relations where profit maximisation is not the only driving ethos.^17^ In the food system, for example, a growing movement of food cooperatives, urban agriculture and community-supported agriculture, operating under public interest and sustainability objectives, are demonstrating viable food economies supporting equitable access to healthy food, fairer livelihoods for producers and environmental sustainability.^18-20^ By underpinning market exchanges with food’s inherent value to our well-being, community life and environmental stewardship, alternative food economies are challenging neoliberalism’s conception of goods and services as ‘neutral,’ recognising value not captured in current supply, demand and price relations.^18,21^

Progressive economist, Kate Raworth, has set out what she calls doughnut economics – the sweet spot for humanity with zero shortfalls in the social foundation (eg, food, income, education) while simultaneously not overshooting our ecological ceiling along any of the planetary boundaries (eg, biodiversity loss, fresh water, ozone depletion). She lays out seven principles for a new economics including: (1) developing to meet the needs of all within the means of the planet; (2) recasting the role of the state, market, society, household and commons in an embedded economy; (3) shifting away from rational economic actors to social and adaptable citizens; (4) embracing complexity, systems and evolutionary thinking; (5) pre-distributing wealth and power rather than re-distributing income and resources; (6) moving towards a circular economy; and (7) becoming growth agnostic.^22^ These seeds of paradigm change are just the few we are familiar with. The 2008 global financial crisis exposed the failures of neoliberalism, while the rapid advance of climate change has put a ticking clock on our chance for real change to carry on as a species. New ideas are cropping up all over, we suggest that public health participate in and amplify conversations that make real space for the NCD agenda rather than trying to squeeze it into the neoliberal one.

## How Do We Achieve Paradigm Change?

While the emergence of neoliberalism is located around the late seventies to early eighties, it actually dates back to 1938, when a group of intellectuals gathered in Paris to critique the political economy narratives of their day: socialism, fascism, and Roosevelt’s New Deal policies in the United States. Ideas from this meeting – that social democracy and the welfare state would crush individualism and ultimately lead to totalitarian regimes^23^ – resonated with many, including wealthy individuals eager to free themselves from tax and regulation. In 1947, the Mont Pelerin Society was formed, comprised of high government officials, Nobel Prize recipients, journalists, economic and financial experts, and legal scholars from all over the world. Over the next several decades the movement’s rich backers funded think tanks and academic positions in key institutions to refine and disseminate the ideology. But it was not until a series of economic crises in the 1970s that devotees of the ideology, many now in positions of power and influence, were able to embed neoliberalism as the best, and only, way to solve the crisis. The election of Margaret Thatcher in the United Kingdom and Ronald Reagan in the United States in the eighties cemented the neoliberal policy package, and through global institutions such as the International Monetary Fund, the World Bank and the World Trade Organisation, neoliberal policies soon began to spread the world over.^24^

It is now time for a broad constituency of actors including health, environmental and civil society to write the economic paradigm for the future predicated on protecting human and planetary health. The public health community has an important role to play in building inclusive coalitions of change across this constituency.^25^ We are under no illusions, this will be a Herculean challenge. A new economic paradigm will have to be cultivated in the face of powerful resistance from corporate and financial elites who have promulgated and benefited from the neoliberal economic regime and occupy positions of considerable political influence. What’s more, worsening global ‘syndemics’ such as NCDs, climate change and rampant inequality mean we do not have the luxury of four decades to incubate a new economic paradigm. Lencucha and Thow have started a critical conversation for public health actors – that the types of policies we need are not just hindered by powerful actors, but by powerful ideas. We view this as an important opening. While public health may never be able to compete with the likes of Nestle or Unilever on money and resources, our strength has always been our numbers. Opening up the competition over discursive power, may just be a stage we can compete on.

## Ethical issues

Not applicable.

## Competing interests

Authors declare that they have no competing interests.

## Authors’ contributions

AS and SG contributed to initial ideas and discussion. AS prepared the initial draft. Both authors provided critical feedback and contributed to the final manuscript.
